# Nurse managers’ perspectives on working with everyday ethics in long-term care

**DOI:** 10.1177/0969733020935958

**Published:** 2020-07-15

**Authors:** Siri Andreassen Devik, Hilde Munkeby, Monica Finnanger, Aud Moe

**Affiliations:** Centre of Care Research, Norway; 1786 Nord University, Norway; 1786 Nord University, Norway; Centre of Care Research, Norway; 1786 Nord University, Norway

**Keywords:** Content analysis, ethics support, management, municipal healthcare services, nursing

## Abstract

**Background:**

Nurse managers are expected to continuously ensure that ethical standards are met and to support healthcare workers’ ethical competence. Several studies have concluded that nurses across various healthcare settings lack the support needed to provide safe, compassionate and competent ethical care.

**Objective:**

The aim of this study was to explore and understand how nurse managers perceive their role in supporting their staff in conducting ethically sound care in nursing homes and home nursing care.

**Design and participants:**

Qualitative individual interviews were performed with 10 nurse managers with human resources responsibilities for healthcare workers in four nursing home wards and six home nursing care districts. Content analysis was used to analyse the data.

**Ethical considerations:**

The Norwegian Centre for Research Data granted permission for this study.

**Findings:**

The analysis resulted in seven subcategories that were grouped into three main categories: managers’ perception of the importance of the role, managers’ experiences of exercising the role and managers’ opportunities to fulfil the role. Challenges with conceptualizing ethics were highlighted, as well as lack of applicable tools or time and varying motivation among employees.

**Discussion:**

The leaders tended to perceive ethics as a ‘personal matter’ and that the need for and benefit of ethical support (e.g., ethics reflection) depended on individuals’ vulnerability, attitudes, commitment and previous experiences. The managers did not seem to distinguish between their own responsibility to support ethical competence and the responsibility of the individual employee to provide ethical care.

**Conclusions:**

Our findings suggest that nurse managers need support themselves, both to understand and to carry out their responsibilities to foster their staffs’ ethical conduct. Supporting staff in conducting ethically sound care requires more than organizing meeting places for ethical reflection; it also requires greater awareness and understanding of what ethical leadership means.

## Introduction

Today’s healthcare environment has made it difficult for nurses and other healthcare workers to practise with integrity amid the complex moral choices and pressures they confront.^1^ New technology, various and increasing demands, limited resources and conflicting values create continuous ethical issues.^2^ In Norway, municipal long-term care (nursing homes and home nursing care services) are particularly challenged by more and sicker patients because of the restructuring of healthcare services^3^ along with an increasingly elderly population.

Together, ethical competence and responsibility form the basis for healthcare workers’ ethical actions.^4^ However, several studies have concluded that nurses across various healthcare settings lack the support needed to provide safe, compassionate and competent ethical care, with resultant moral distress.^5–10^ Poor performance, burnout and leaving the profession are also reported.^11^

Nurse managers are significant stakeholders in the running of the organization, the quality of care and the welfare of the staff in nursing homes and home health care services. They are responsible for clarifying the organization’s mission, vision and values^12^ and are expected to continuously ensure that ethical standards are met, while supporting healthcare workers’ ethical competence.^13^ They also play an important role in promoting practices that respect patients’ humanity and dignity and ensuring patient safety.^14^ Ethical leadership requires responsiveness to both practitioners and the contextual system in which leaders and staff work; therefore, leaders must receive and provide support to enhance the capacity of practice and stimulate reflections about everyday ethics.^15^ The leadership style exhibited is shown to have a direct and unique impact on the working atmosphere,^16^ but nurse managers often feel uncertain about how to lead in ethical dilemmas.^17^ Their leadership style is often orientated to maintenance and focused more on ‘doing the job’ than on managing decision-making in ethical dilemmas.^18^

During the period from 2007 to 2015, nearly half of the Norwegian municipalities participated in a national ethics project that aimed to enhance ethical competence among staff in community healthcare.^19^ Facilitator-led ethical reflection in groups was the most applied intervention among the participating municipalities.^20^ Staff indicated that group reflection on ethics contributed significantly to perceived quality and competence and was considered a meaningful activity promoting better handling of ethical dilemmas.^21^ However, organizational instabilities in community healthcare may hinder achievements,^20^ and evaluations in 2014^22^ showed that only a minority of the municipalities had implemented systematic ethical reflection. Among promoting factors, health personnel find that organizational support – especially from department management – is an important driving force, in that someone organizes and actively expects results from the ethics reflection.^20^

Several studies focus on the ethical problems nurse managers may encounter^17,23^ and their ethical competence related to dealing with such problems.^24^ However, less is known about how leaders perceive and attend to their role in supporting their staff and promoting ethical conduct in their organizations.^25,26^ The aim of this study was therefore to explore and understand how nurse managers perceive their role in supporting their staff in conducting ethically sound care in nursing homes and home nursing care.

## Method

### Design

A qualitative approach was chosen to get insight into nurse managers’ experiences, beliefs, notions and thoughts in order to answer the research question.^27^ Semi-structured interviews^28^ were conducted individually with the participants.

### Setting and participants

The study was carried out in nursing homes and home nursing care services in three municipalities in mid-Norway from April to October 2018. The municipalities had participated in the national ethics project conducted in 2013–2014. Administrative healthcare leaders distributed information and invitation letters to nurse managers with human resources responsibilities for healthcare workers in nursing home wards and home nursing care districts. Ten nurse managers, nine women and one man, consented to participate. Their ages ranged from 41 to 62 years, and they had been working as managers for 6 months to 19 years (median 7 years). They represented ward-level management, with responsibility for 22 to 53 employees each per ward/district, including registered nurses, auxiliary nurses, social educators, apprentices and assistants. Four of the participants managed nursing home wards in the areas of geriatric, psychiatric, dementia, palliative, acute and rehabilitation care. The remaining six were district managers in home nursing care covering elderly care services in both private homes and assisted living facilities.

### Data collection

The data were collected via semi-structured interviews conducted by the first author. Initially, the managers were asked to describe staffing, daily care and patient groups on their wards. They were also asked to share how they worked to prevent sick leave and how they followed up with staff in terms of work ability and the quality of services they provided. Then, they were asked to describe any experiences with interventions to enhance ethical competence among staff and their views about their own roles in that regard. Finally, they were asked if exception reports could apply to violations of ethical standards. Background information like gender, age, occupation, years in the position, staff composition and their assessment of workload was also collected. The participants received the questions in advance, and the interviews took place at their offices. The interviews lasted between 48 and 70 minutes and were audiotaped and transcribed verbatim.

### Data analysis

The interview texts were analyzed by qualitative content analysis.^29,30^ All interviews were read several times to get an impression of the whole text. When searching for meaning units (words, phrases or paragraphs) that could be connected to the aim, each interview was seen as the unit of analysis.^30^ The scope was on the manifest content, which is the visible, obvious component of what the text said.^31^ The identified meaning units were condensed and then coded to reflect the content they represented. In the next step, the condensed meaning was compared across all the interviews in a search for patterns of similarities and differences. The condensed meanings were then sorted and abstracted into seven subcategories and three categories.

### Ethical considerations

This study was evaluated and approved by the Norwegian Centre for Research Data (project no. 59444). The participants received both oral and written information about the study purpose and procedure and gave their signed consent before the interviews started.

## Findings

The analysis resulted in three categories: (1) managers’ perception of the importance of the role, (2) managers’ experiences of exercising the role and (3) managers’ opportunities to fulfil the role, with seven pertaining subcategories. An overview of the findings is shown in Table 1.

**Table 1.** Overview of categories and subcategories.

Categories	Managers’ perception of the importance of the role	Managers’ experiences of exercising the role	Managers’ opportunities to fulfil the role
Subcategories	*Ethical challenges faced by the staff* *The staff’s handling of ethical challenges*	*The value of working with ethics* *Barriers to ethics work*	*Leadership in ethics work* *Arenas for ethics work* *Responsibility in ethics work*

### Managers’ perception of the importance of the role

The managers’ perception of the role’s significance was seen as an expression of how they perceived employees’ need for ethical support. This was based upon what they saw as ethical challenges at their workplaces and how their staff managed the situations. This category included the following subcategories: ethical challenges faced by the staff and the staff’s handling of ethical challenges.

#### Ethical challenges faced by the staff

Independent of the healthcare setting, all of the respondents noted that their work environments were characterized by heavy workloads and shortages of time and personnel. They could see that their staff were struggling to keep up with duties and the quality standard of care to patients with increasingly advanced healthcare needs. The conflict between giving optimal care and the manageable reality was seen as ethically challenging for several of their employees. One manager in home nursing care said,

The staffing is marginal…I can see that they are running, especially in the evenings and weekends…There is a gap between how they want to do things and what the resources allow for…Sometimes I just have to tell them that they have set the bar too high.

In home nursing care, some managers explained that patients’ demands appeared endless and caused much frustration among staff when they were not able to comply with them. Other times, they observed that staff felt anguished about whether they were doing enough for the patients, for example, when the agreed service concerned administering eye drops, while the patient really wanted assistance with fetching fire wood. The managers also recognized that their staff had to show more consideration to user involvement and relate to a ‘new’ kind of patient, as one manager in a nursing home explained,

Times have changed…We have to face new demands, new questions, a new type of family caregiver, and adapt to more user involvement…We do not just do as we like anymore, we have to change too…and that is often difficult. Perhaps there is a need to stop for a while, think about right and wrong.

Disagreements between staff and patients or between staff and family caregivers occurred often and caused much discussion in the charge room. Sometimes the leaders were involved in these discussions. One manger in a nursing home said,

Family caregivers can be very coercive in trying to get things done, no matter if the patient has resisted. So…what is best for the patient? And who is to decide that?

Conflicting views about patient needs or how to provide the best care were also seen among different healthcare workers. Several examples were given of situations where employees felt conflicted between considerations for the patient and loyalty to a colleague. Sometimes, there was disagreement about the best care in a specific situation, and other times employees struggled with standing up for patients who were exposed to insulting behaviour from colleagues.

The leaders continually returned to the fact that ethics and ethical challenges were hard to grasp or define. Some leaders’ impressions were that this concept rather was referred to as frustrations, sometimes concerning lack of resources, other times concerning challenges related to professional behaviour.

#### The staff’s handling of ethical challenges

In general, most of the managers believed that their staff dealt adequately with everyday ethics. Although they admitted that their knowledge about the staff’s handling and coping on an individual level varied, they trusted that both education and moral discretion were active and protective factors. One manager in a nursing home stated,

Actually, it shouldn’t be necessary to give directions for working with ethical issues…Ethics is a natural part in our everyday practice – it should be in our backbone, and I really believe that the staff reflects a lot, for example, in the accounts given between shifts…but, it might be more or less coincidental, of course.

There were several examples of employees who turned to their leaders with their problems, but also of staff supporting each other. The managers also perceived that their employees managed differently and that some found their jobs so mentally exhausting that they had to get a sick note. One manager in home nursing care said,

Some are always tidy and neat in everything they do, while others go more easy on it, and some are more personally influenced…I think it lies in their personality, it is not because of their education. It is difficult…but perhaps we need to learn that good enough is good enough.

### Managers’ experiences of exercising the role

The managers’ experiences of ethics work dealt mainly with participation in the national ethics project. Despite the fact that the project had survived to a small extent, the managers had clear perceptions of both the value of ethics work and what prevented its implementation.

#### The value of working with ethics

All the managers, except one, could recall memories about taking part in the national ethics project, but some of them had not been leaders at that time. Ethical reflection in groups of colleagues, led by a facilitator, was the main activity in which they had been involved. The organization of ethical group reflection varied, which meant that some groups were open to participants based on their willingness and availability at the time of the session, while other groups were carried out with the same, permanent participants each time. Common to all was the designation of facilitators who had received special training to lead the group reflections. Some of the managers believed that participation had been very useful for a handful of people, especially for the facilitators.

Despite no longer having the full benefit of the national ethics project, all of the managers upheld the need for and the value of working with ethics. Based upon their impressions of the everyday ethics on their wards, the leaders emphasized the benefits of putting ethics on the agenda. Although ethics group reflection was not systematized, they often witnessed fruitful discussions about ethical challenges when the staff met for lunch or had mid-day report. One manager in a nursing home said,

It probably helps just to talk about it, and in a way becoming reassured in that what they do is actually right. One really needs this colleague support, because there is not always any obvious right answer.

In addition to getting confirmation about the validity of one’s thoughts and decisions, the leaders also meant that discussing ethics helped illuminate the staffs’ scope of action and increased their action skills. Reflection on practice was also seen as a means to increase feelings of security among the staff and their resilience at work in regard to both presence and caring abilities. One manager in home nursing care stated,

To me, ethics is highly connected to the work environment and the security of being who you are…and if we manage to convey that our work environment is open for whatever needs to be discussed and that we tolerate differences, then we can strengthen our feelings of unity and even contribute to recruitment.

Several of the managers also gave examples of employees who had turned to them and presented dilemmas they needed to discuss. One manager in nursing home said,

She had lost sleep and was very troubled by this situation. If she hadn’t opened up, I think she’d be on sick leave now.

The same manager added,

When you recognize that this [ethical dilemma] is good to talk about, because it makes you think a little different and feel other kinds of feelings…then, it makes you healthier – then, it is helpful. The choice will be correct anyway – but one must argue for it and feel that afterwards one actually made reflections that led to the decision - then the choice is right for you in any case.

Some of the managers in nursing homes pointed to the transfer value regarding ethics, when working within the framework of person-centred care. One manager stated,

To be able to say that we work [in a] person-centred [way], we must have a common understanding of the values. In person-centred care, one must also try to investigate the patient’s behaviour and find out why…the patient struck [someone], for instance. In my opinion, [a] person-centred approach automatically strengthens ethics reflection and vice versa…

Increased awareness of ethical issues appeared to be the most highlighted advantage of the national project. Simultaneously, some managers mentioned that their personal interest and competence in relation to ethics was relevant. One manager in home nursing care was convinced that her own interest and education in ethics, as well as her former experience as an ethics facilitator, was crucial to her ability to put ethics on the agenda. She said,

I’m passionate about it. This is important to me both as a starting point and to make it become a present element in the everyday life on the ward.

The managers rarely found that exception reports could apply to violations of ethical standards. However, putting ethics on the agenda through exception reports was discussed as valuable for raising employees’ ethical awareness.

#### Barriers to working with ethics

Difficulties in prioritizing time for ethical reflection emerged as a huge barrier for the leaders. They realized that this was especially true for the nurses, who were seen as the work team leaders and were already fully occupied with a variety of both user-oriented and administrative tasks that required time and attention. They believed that ethical reflection was often referred to periods of less bustle, when the presence of staff was stable or when someone randomly decided that they should carry it out.

The managers also noted little engagement among employees as they tried to bring them together for a round of ethical reflection. One of the managers in home nursing care stated,

I find it difficult to be the one to create motivation among employees…perhaps it would have been easier if we had good tools. We manage it in the project we are currently working on, to prevent coercion. In that project, we have a study circle and specific tools…videos, workbook…it becomes a practical act in any way.

Only one manager said that ethical group reflection, modelled on the national project, was implemented as a routine at their workplace today. Overall, the managers expressed resignation when describing efforts to implement ethical reflection. One manager in home nursing care said,

We have talked about deciding that the first Wednesday every month should be set aside for ethical reflection, but it has been deprioritized. Ethics is extremely important, but somehow we are not able to organize it or be systematic about it. It sort of flows off…

The managers talked most about the difficulties they experienced in continuing the ethics work launched by the national project.

One manager in home nursing care said,

The project had good intentions, but these were abandoned when lack of time required emphasis on other priorities. It is the direct user-oriented contact that is in focus – and they (the staff) are very good at that.

Another manager in home nursing care added,

The facilitator really tried – but it got a little difficult, alienated – not everyone who understands what this is…Perhaps it is hard to be conscious of what these conflicts, we feel inside, mean or are? I don’t think the staff experienced it [as] so very helpful or recognizable, no…it became too difficult. Everyone should try to find the ethical dilemma we should discuss – but an ethical dilemma was difficult to define…

Other managers talked about difficulties when the sessions of reflection took place outside of working hours or created logistical problems with shift work. Several of the managers also felt that the outcome of the project relied much upon the person, his or her personal engagement, whether it concerned facilitators, staff or leaders. In addition, they felt that the healthcare sector is characterized by the fact that new projects are constantly coming up and one is not ready to implement anything until one has started something new.

### Managers’ opportunities to fulfil the role

The managers identified several important conditions for working with ethics on their units, which were sorted into the following subcategories: leadership, arenas and responsibility.

#### Leadership in ethics work

All managers stated that they strived to be close to their employees and to foster open and positive relationships. In hiring, they tried to get to know the employees and regularly followed up with appraisal meetings. Finding time for these meetings was often complicated by busy schedules. However, appraisal meetings were highlighted as opportunities to gain knowledge about the employees’ qualities and values, as well as their shortcomings and self-awareness. One manager in a nursing home said,

It is not necessarily the number of personnel on the shift that is decisive, but who they are. Do they have the right qualities? Are they aware of how they react and act on others?

They were also concerned that the staff should feel that there was a low threshold for contacting managers. Overall, the leaders expressed a strong desire to be supportive and involved with the employees, but admitted that hectic everyday life could make it difficult to achieve these visions. At the time of the interview, several of the managers were in the midst of downsizing and reorganization processes. Few of the leaders talked explicitly about their leadership style, but some mentioned value-based leadership, relational leadership and trust-based leadership as inspirational approaches. When it came to measures to stimulate employees’ presence and working abilities, the leaders agreed that clarity regarding duties and rights was the most effective. Some gave examples of honouring employees who made special efforts. Others discussed episodes where they used negative examples of employees who had shown poor ethical judgment to enhance shared learning at staff meetings.

#### Arenas for ethics work

The managers identified various arenas or meeting points that were suitable and potentially available for ethics work or ethical reflection. These meeting points included accounts between shifts, mid-day reports, coffee breaks, reflection meetings on particular patient cases, staff meetings and professional meetings among nurses or other professionals. Social meetings outside working hours were also seen as beneficial for teambuilding and raising feelings of security among the staff. However, most of the available arenas were used for dealing with urgent practical issues or day-to-day challenges. Only one manager in home nursing care said that ethical reflection was scheduled to replace the mid-day report once a week.

#### Responsibility for ethics work

The responsibility for working with ethics materialized primarily through attempts to set aside time for ethical reflection and to handle reported nonconformities. The managers said that the focus on ethics was significant both in national and local healthcare policy plans and in meetings with other healthcare leaders in the municipality. Still, the translation of good intentions into practical action seemed problematic. One manager in home nursing care explained,

Ethics is seen as important in our strategies for enhancement of the staff’s competence and we discuss it a lot at our leader meetings, but the formulations are vague and hard to translate into actual and common practice…I think we all try to do what suits our local wards/units best. And as leaders, we are also different persons holding different views…

Other managers indicated that the responsibility for the ethics work was unclear, exemplified by one manager in a nursing home,

We talk a lot about ethics, but there are no guidelines for me as a leader, not that I know of. Actually, I wish we could have our own ethical reflection meetings in the leadership group…I think it is possible.

On the one hand, the managers argued that ethics permeated all of the activity on their wards and that they were constantly working on it. On the other hand, they admitted that more systematic work would have been desirable.

## Discussion

The managers in this study experienced ethics work as valuable but challenging. They saw value in the sense that supporting ethical competence helps secure quality of care and also enhances the individual employee’s coping skills and ability to live up to his or her ethical standards. However, working practically with ethics was experienced as difficult and not so evident. Organizational obstacles and time constraints were offered as explanations, as were lack of applicable methods and difficulties in communicating about ethics. Our findings indicate that leaders had a somewhat distant relationship to ethics and drew more attention to the individual than the organizational and managerial responsibility for promoting ethical conduct on their wards. An understanding of ethics as a ‘personal matter’ seemed to pervade their experiences. First, the managers recognized that employees’ handling of ethically difficult situations could depend on the vulnerability of the individual employee. Second, ethical competence was seen as a matter of course, and an individual and personal responsibility of anyone working within patient care. Third, implementation of ethics was perceived to be highly influenced by individuals’ (both employee’s and leaders’) attitudes, commitment and previous work experiences. The managers did not seem to separate their own responsibility for supporting ethical competence from the responsibility of the individual employee to provide ethical care.

The leaders’ impressions of what was ethically challenging for the staff correspond to ‘everyday ethics issues’ (e.g., patient autonomy and offensive behaviour) and ‘big ethical issues’ (e.g., life-or-death matters and treatment choices).^32^ Still, the leaders placed most concern on the challenge of the individual employee’s personal morale when optimal care was prevented by organizational factors like work instructions and lack of resources. In such cases, the ethical challenge or frustration was explained by variations among employees’ personalities, and hence, ethics became a personal and private matter. Ethics seen as something intimate and confidential, only shared between an individual and his or her conscience,^33^ also implies that the individual is left alone with the responsibility. Yet, more research demonstrates nurses’ distress and fear of becoming superficial and indifferent when heavy workloads require prioritization and may prevent satisfactory and morally good nursing^34,35^ and calls for more attention to collective and managerial responsibility.^33^

The managers’ statements were normative when talking about the value of ethics work. However, how to make ethics understandable and a subject for reflection was not so self-evident. Challenges with conceptualizing ethics were highlighted, as well as the lack of tools or time and varying motivation among employees. The latter could also vary among leaders. One impression from the interviews was that some leaders regarded these challenges as beyond their control. In addition, they seemed to rely on the idea that employees nonetheless reflected a lot during the reports.

Although the importance of ethics seemed indisputable, and arenas for ethics reflection were identified, the managers confirmed previous research indicating that attention to ethics may lose out to other pressing tasks in a care environment.^18^ For some leaders, their solution was to emphasize ethics by focusing on person-centred care. Others talked about the importance of recruiting employees with good values, while all stated that they made efforts to set aside time for ethical reflection. Overall, the managers seemed to apply an instrumental approach to ethics work, and they acknowledged that they were not as close to the day-to-day staff as they wanted to be.

Without being explicit about it, the leaders mentioned that they were inspired by various leadership styles. According to Brown and Treviño,^36^ moral managers make ethics explicit by communicating values and intentionally role-modelling ethical behaviour. Implicitly, it is not enough to provide directives for good moral care; the leader must also be able to demonstrate it. Ethical leadership is increasingly regarded as a separate and proactive management style^37^ that is characterized by the demonstration of appropriate conduct through personal actions and interpersonal relationships.^38^ Equally important is being able to identify ethical aspects in a given situation.^39^ Values and morals are linked to personal and individual circumstances. At the same time, this is shaped by education, professional guidelines, society and the specific context in which ethics take place. Ethical issues concerning context that do not necessitate immediate medical actions towards patients are often overlooked.^33^ Examples of such issues may be the staffing situation, lack of time and limiting work instructions. The managers in this study did recognize organizational factors to be ethically relevant, but only when situations contradicted the individual employee’s caregiving standards. Perhaps the managers, deliberately or not, chose to understand these as frustrations employees had to live with because the managers themselves felt powerless. However, when dealing with organizational ethics, involvement from leaders is a must,^32^ and the first step may be to recognize such frustrations as both ethically significant and as a collective concern within the organization. Because personal values are too variable to serve as guidelines, nurse managers should facilitate discussions, convey clear and transparent solutions, and serve as role models in solving ethical problems.^12,15,26^

Our findings suggest that nurse managers need support themselves, both to become aware of and to carry out their responsibilities to support their staff’s ethical conduct. Supporting staff in conducting ethically sound care require more than organizing meeting places for ethical reflection; they also require greater awareness and understanding of what ethical leadership means. Ethical reflection groups for leaders and development of educational programs for ethical leadership can be suggested as concrete implications from this study. The findings also point at challenges to ethics work in organizations that should be explored in further studies.

This study was carried out in a limited geographical area and included a relatively small sample of nurses who had different experiences in management. The transferability of our findings is facilitated by the participants’ rich descriptions, the context and a significant proportion of quotations from the interviews.

## Conclusion

Our findings showed that nurse managers experienced ethics work to be valuable but also challenging. Challenges with conceptualizing ethics were highlighted, as well as lack of applicable tools or time and varying motivation among employees. They tended to view ethics as a ‘personal matter’, and that the need and benefit of ethical support (e.g., ethics reflection) depended on individuals’ vulnerability, attitudes, commitment and previous experiences. The managers did not seem to distinguish between their own responsibility for supporting ethical competence and the responsibility of the individual employee to provide ethical care. Like their staff, nurse managers need to be more aware of ethical aspects in given situations. They also need support to understand and practice ethical leadership, which can be facilitated through ethical reflection for leaders and the development of the training program.
